# The role and mechanism of mitochondrial functions and energy metabolism in the function regulation of the mesenchymal stem cells

**DOI:** 10.1186/s13287-021-02194-z

**Published:** 2021-02-17

**Authors:** Wanhao Yan, Shu Diao, Zhipeng Fan

**Affiliations:** 1 Laboratory of Molecular Signaling and Stem Cells Therapy, Beijing Key Laboratory of Tooth Regeneration and Function Reconstruction, Capital Medical University School of Stomatology, Beijing, 100050 China; 2 Research Unit of Tooth Development and Regeneration, Chinese Academy of Medical Sciences, Beijing, China; 3 Department of Pediatric dentistry, Capital Medical University School of Stomatology, Beijing, 100050 China

**Keywords:** Mitochondria, Energy metabolism, Reactive oxygen species, Mitochondrial transfer, Mesenchymal stem cells

## Abstract

Mesenchymal stem cells (MSCs) are multipotent cells that show self-renewal, multi-directional differentiation, and paracrine and immune regulation. As a result of these properties, the MSCs have great clinical application prospects, especially in the regeneration of injured tissues, functional reconstruction, and cell therapy. However, the transplanted MSCs are prone to ageing and apoptosis and have a difficult to control direction differentiation. Therefore, it is necessary to effectively regulate the functions of the MSCs to promote their desired effects. In recent years, it has been found that mitochondria, the main organelles responsible for energy metabolism and adenosine triphosphate production in cells, play a key role in regulating different functions of the MSCs through various mechanisms. Thus, mitochondria could act as effective targets for regulating and promoting the functions of the MSCs. In this review, we discuss the research status and current understanding of the role and mechanism of mitochondrial energy metabolism, morphology, transfer modes, and dynamics on MSC functions.

Mesenchymal stem cells (MSCs) are multipotent stem cells with self-renewal abilities and multi-directional differentiation potentials [1]. They also show paracrine and immune regulation functions that present great clinical application prospects in injured tissue regeneration, functional reconstruction, and cell therapy [2, 3]. Initially, the MSCs were first isolated from the bone marrow and then from other tissues such as adipose, umbilical cord, and dental pulp. The in-depth studies on the utility of the MSCs have reported that they show therapeutic effects in bone loss diseases [4], tooth and periodontal tissue regeneration [1], liver injury [5], and nerve injury [6]. The functions of the MSCs are regulated by growth factors, inflammatory mediators, extracellular environment, cell transduction signals, and cell metabolism [7]. However, the direction differentiation of the transplanted MSCs is difficult to control, and the cells are prone to ageing and apoptosis in the local damaged tissues; this leads to an impairment in their functions and makes it difficult for the damaged MSCs to effectively exert their functions and achieve the ideal regeneration and reconstruction abilities [8]. Thus, improving the functions of the MSCs, such as by promoting their directional differentiation, inhibiting their ageing and apoptosis, and promoting local tissue regeneration under clinical conditions, is of major relevance in stem cell therapies.

Organelles in cells mainly include endoplasmic reticulum (ER), Golgi apparatus (GA), ribosome, mitochondria, and so on. The ER and ribosome acts as a protein synthesis factory, which is involved in the production, folding, modification, maturation, quality control, and degradation of approximately one third of cellular proteins and makes certain that only properly folded proteins can be transported to their intra-cellular or extracellular sites [9]. While the GA is a processing and dispatching station, whereby newly synthesised soluble and transmembrane proteins, as well as lipids, are sorted for subsequent transport to the cell surface, secretory granules, or the endosomal system [10]. However, these biological processes are closely related to the utilisation of adenosine triphosphate (ATP). Mitochondria are the primary site of oxidisation of carbohydrates, fats, and amino acids to produce ATP [11]. Thus, they are important organelles for cell energy metabolism. In recent years, the studies have reported that the remodelling of mitochondria has been observed during MSC differentiation and maintenance [12, 13]. The mitochondrial morphology, distribution, transfer, biogenesis, dynamics, and mitophagy are crucial to maintain the homeostasis, and regulate the fate of the MSCs. Thus, mitochondria play a major role in regulating stem cell self-renewal, multi-directional differentiation, ageing, apoptosis, and immune regulation [14, 15]. In addition, the mitochondrial energy metabolism can regulate the functions of the stem cells through many mechanisms, including glycolysis, redox reaction in oxidative phosphorylation (OXPHOS), energy metabolism process conversion, change in mitochondrial membrane potential (MMP), production of intra-cellular reactive oxygen species (ROS), and oxidative stress [16, 17]. As mitochondrial functions and energy metabolism are essential for regulating various properties of the MSCs, these processes may provide an effective way to regulate the functions of the MSCs. Thus, in this review, we highlight the current studies and discuss the effects of mitochondrial functions and energy metabolism on the functions and therapeutic potential of the MSCs.

## Mitochondrial function and energy metabolism pathways

Mitochondria are double membrane-bound organelles that are responsible for energy generation in cells by the oxidation of carbohydrates, fats, and amino acids. They are semi-autonomous organelles with their own genetic material, genetic system, and a limited genome [18]. Further, their diameters are approximately 0.5–1.0 μm. In addition to providing energy for cells, the mitochondria are also associated with several essential metabolic pathways, such as the tricarboxylic acid cycle (TCA cycle), fatty acid β-oxidation, and single carbon cycle. The metabolites produced by these pathways can also be used as retrograde signals to regulate the function of the MSCs [19]. Moreover, the mitochondria possessed by different MSCs vary in size, number, and appearance. The number of mitochondria depends on the metabolic level of the cell [20, 21]; consequently, the cells with a high metabolic activity have more mitochondria.

The main pathways of mitochondrial energy metabolism in cells are glycolysis, TCA cycle, and OXPHOS. Glycolysis and the TCA cycle produce reduced nicotinamide adenine dinucleotide (NADH), reduced flavin adenine dinucleotide, and other energetic molecules; while OXPHOS uses these substances to reduce O_2_ and release energy to synthesise ATP. If a cell is in a hypoxic environment, it switches to anaerobic respiration; at this time, the pyruvate produced by glycolysis no longer enters the TCA cycle in the mitochondria, but continues to react and is finally reduced by NADH into fermentation products, such as ethanol or lactic acid, rather than ATP [22]. Mitochondria also play vital roles in amino acid, fatty acid, and steroid metabolism. Moreover, malonylation, succinylation, and glutarylation of the amino acid lysine utilise these substrates of the mitochondrial fatty acid and amino acid metabolism [23]. Different stem cells, as well as different biological processes of the same cell, can undergo a shift in energy metabolism [24, 25]; thus, mitochondria can regulate the function of MSCs by changing their energy metabolism pathways.

Furthermore, mitochondria are crucial organelles responsible for signal transmission in the MSCs. They play an important role in regulating cell signals produced by ROS [17], calcium homeostasis [26], and membrane potential [27]. Mitochondria are the main source of intra-cellular ROS production as mitochondrial OXPHOS produces large amounts of ROS as a by-product. The ROS include O_2_^−^, H_2_O_2_, OH^−^, and LOOH that can provide O_2_ free radicals in biochemical reactions and have strong biological activities. Further, the NADH-CoQ oxidoreductase (complex I) and ubiquinone-cytochrome C oxidoreductase (complex III) in the respiratory chain of the mitochondrial inner membrane can release electrons to produce O_2_^−^, which is the precursor of most ROS. Superoxide dismutase (SOD) can also be activated to produce H_2_O_2_, catalase, or glutathione peroxidase. Moreover, other metabolic intermediates including 2-ketoglutarate dehydrogenase, pyruvate dehydrogenase, and glycerol-3-phosphate dehydrogenase are also involved in the upregulation of ROS production in the MSCs [28]. The production of ROS at a normal level is essential to maintain the activity of the MSCs; however, during oxidative stress in cells, the ROS levels increase dramatically and may severely damage the MSCs [17]. Therefore, eliminating high ROS levels and its corresponding side effects in mitochondria, by maintaining a normal physiological level of ROS, is of great significance to enhance the activity of the MSCs. Finally, key factors such as hypoxia-inducible factor-1α (HIF-1α), PPARγ coactivator-1α (PGC-1α), sirtuin (SIRT), superoxide dismutase 2 (SOD2), adenosine 5′-monophosphate-activated protein kinase (AMPK), and uncoupling protein (UCP) also play a regulatory role in the function of the MSCs.

## The role of mitochondrial morphology and distribution in the regulation of MSC functions

During low energy demand, the mitochondria are generally small, fragmented, round, and with under-developed cristae; while during high energy demand, the mitochondria transform into an elongated shape with well-developed cristae. These ultimately affect the number of mitochondria, cell metabolism, and mitochondrial activity [15]. Thus, the morphology and distribution of mitochondria can be used as main characteristics to identify the MSC differentiation. For instance, the osteogenic differentiation of bone marrow mesenchymal stem cells (BMSCs) is accompanied by the development of mitochondrial cristae [19]. Moreover, the arrangement of the mitochondria is different before and after the MSC differentiation. Further, the perinuclear arrangement of the mitochondria may be one of the characteristics of undifferentiated MSCs, as mitochondria are mainly concentrated around the nucleus in the undifferentiated MSCs, and uniformly distributed in the cytoplasm in the differentiated MSCs [29]. Moreover, the area ratio of mitochondria to cytoplasm also increases during differentiation [30]. In addition, these morphological changes can affect the differentiation function of the MSCs. One study reported that carbon black, a representative of carbon toxicants, inhibits the osteogenic differentiation of the BMSCs by impairing the morphology and integrity of their mitochondria. During this treatment, the integrity of the mitochondrial cristae structure is gradually lost and is accompanied by mitochondrial swelling, abnormal density, and vacuolar degeneration [31].

## The role of mitochondrial transfer in the regulation of MSC functions

The MSC-mediated transfer of mitochondria (MitoT) refers to the transfer of mitochondrial DNA (mtDNA) from donor MSCs to recipient cells with abnormal mitochondrial function, and through co-culture to restore the normal mitochondrial functions in the recipient cells. MitoT can modulate the bioenergy of the receptor cells by regulating their mtDNA replication, maintaining copy number, and regulating mitochondrial dynamics and the cellular processing required to maintain an intra-cellular mitochondrial homeostasis [15]. When the induced pluripotent stem cell (iPSC)-MSCs are co-cultured with damaged cells or tissues, the mitochondrial respiration and ATP levels are upregulated, and the oxidative damage is reduced [32]. Further, MSC regulation has been used in the treatment of an increasing number of animal disease models through mitochondrial transfer, and paracrine, exosome, and directed differentiation [33]. At present, there are many organelle-based therapies for immune diseases, and many evidences show that the functional status of the immune-competent cells is related to their metabolic statuses [34]. Therefore, MitoT can be further studied to discover its effects on the receptor sites, and it may serve as a potential treatment.

MitoT can occur and treat different diseases through the formation of tunnelling nanotubes (TNTs), gap junctions (GJs), formation of extracellular vesicles (EVs), cell fusion, etc. (Table 1). Among them, TNTs are the most common mode to transfer mitochondria. For instance, Jiang et al. reported that MitoT is a ubiquitous inter-cellular transfer mechanism between BMSCs and a variety of ocular cells, such as corneal endothelial cells, retinal pigment epithelial cell lines, and photoreceptor cell lines, and is dependent upon F-actin-based TNTs [35]. Jackson et al. observed that mitochondria transferred from the BMSCs, partially through TNTs, could enhance the phagocytosis of the macrophages in mouse models and thereby ameliorate acute respiratory distress syndrome (ARDS) and sepsis [36]. In addition, the BMSCs could protect target organs from apoptosis through a mitochondrial transfer of TNTs and play a role in the treatment of acute lymphoblastic leukaemia (ALL). Furthermore, reducing the number of mitochondria or using inhibitors such as vincristine (to reduce the mitochondrial transfer) prevent the “rescue” function of the activated BMSCs in the ALL cells, and lead to the apoptosis and death of all targets in the treatment site [37]. Lastly, Luz-Crawford et al. analysed the ability of healthy donor BMSCs to transfer mitochondria to primary CD4^+^ CCR6^+^ CD45RO^+^ Th17 cells and reported that the Th17 cells could absorb mitochondria from the BMSCs through TNTs, and that could affect their immune regulation functions. Further, the mitochondrial transfer to the Th17 cells was impaired when co-culturing with human synovial MSCs (sMSCs) from patients with rheumatoid arthritis (RA) when compared with healthy BMSCs; in addition, this artificial MitoT also significantly reduced the IL-17 production in the Th17 cells, suggesting that a reduced mitochondrial transfer by the RA-sMSCs may be the main reason for the persistence of chronic inflammation in RA synovitis [38]. MitoT can also occur through GJs. Islam et al. reported that gap junctional channels can be formed between the BMSCs and injured alveolar epithelial cells, to facilitate the transfer of mitochondrial-encapsulated vesicles into the alveolar epithelial cells; these vesicles are then ingested by endocytosis to treat acute lung injury [39]. Recent studies have also reported that the role of MSCs is mainly due to the transfer of EVs. EVs are able to transfer a variety of substances, including organelles such as mitochondria, and are thus considered a more feasible candidate for therapy than whole-cell delivery. Such as, the mitochondria in the BMSCs are mainly transferred to CD4^+^ T cells through EVs. The artificial transfer of mitochondria from the BMSCs increases the expression of the mRNA transcripts involved in T cell activation and regulation of T cell differentiation, including *FOXP3*, *IL2RA*, *CTLA4*, and *TGFβ1*, thereby resulting in increased suppressive CD25^+^FoxP3^+^ population. In a graft-versus-host disease mouse model, a MitoT-induced transplantation of human T cells can significantly improve the survival, reduce tissue damage and reduce the infiltration of T-CD4^+^, T-CD8^+^, and T-IFN-γ^+^ expressing cells in organs [40]. Further, the BMSCs can promote the anti-inflammatory function of alveolar macrophages in an ARDS environment through the mitochondrial transfer mediated by EVs; this stimulates the expression of the macrophage phenotype that shows high phagocytosis. Further, the BMSC-derived EVs can also reduce inflammation and lung injury in lipopolysaccharide-injured mice in vivo [41]. Finally, the mitochondria in the MSCs can also be transferred to recipient cells by cell fusion. For example, adipose-derived mesenchymal stem cells (AD-MSCs) co-cultured with cardiomyocytes can transfer mitochondria by cell fusion, and then reprogram the adult cardiac cells towards a progenitor-like state to achieve therapeutic effects [42]. In addition, the MSCs derived from different tissue sources show differences in mitochondrial respiration, donor capacity, and therapeutic effects. For instance, the BMSCs and AD-MSCs have obvious mitochondrial transfer characteristics, while dental pulp stem cells and umbilical cord-derived mesenchymal stem cells (UCMSCs) have apparent aerobic respiratory capacities; subsequently, they transfer the same number of mitochondria and show effective therapeutic effects [20]. In general, the MSCs can transfer mitochondria through various modes to restore the mitochondrial functions in the target cells to rescue the target organ damage, such as an ocular tissue injury, lung injury, and myocardial injury, and play an important role in immune regulation. Lastly, the mitochondria isolated from MSCs can be directly introduced into injured tissues as drugs to mimic the mitochondrial transfer in vivo; this may be a new treatment for diseases and thus warrants the need for future studies [43].

**Table 1:** Mitochondrial transfer modes from different tissue-specific MSCs to recipient cells of different origins

MSC types	Recipient cell	Mode of mitochondrial transfer	Action	References
BMSCs	Corneal endothelial cells, 661W cells, and ARPE-19 cells	TNTs	Ocular tissue regeneration	Jiang D et al., Theranostics, 2020 [35]
BMSCs	Macrophage	TNTs	Enhance macrophage phagocytosis, and thereby improve ARDS and sepsis	Jackson MV et al., Stem Cells, 2016 [36]
BMSCs	Acute lymphoblastic leukaemia cells	TNTs	Protect the target organs from apoptosis	Burt R et al., Blood, 2019 [37]
BMSCs	Th17 cells	TNTs	Affect the immune regulation function of the Th17 cells and promote the acquisition of anti-inflammatory phenotype by pro-inflammatory Th17 cells	Luz-Crawford P et al., Stem Cell Res Ther, 2019 [38]
BMSCs	Alveolar epithelial cells	Gap junctions	Treatment of acute lung injury	Islam MN et al., Nat Med, 2012 [39]
BMSCs, umbilical cord blood mesenchymal stem cells	CD4^+^ T cells	EVs	Involved in T cell activation and reduction of tissue damage in graft-versus-host disease	Court AC et al., S EMBO Rep, 2020 [40]
BMSCs	Mouse alveolar macrophages	EVs	Promote the anti-inflammatory effects of macrophages and express the phenotype of macrophages with high phagocytic functions in an acute respiratory distress syndrome model	Morrison TJ et al., Am J Respir Crit Care Med, 2017 [41]
Adipose-derived mesenchymal stem cells	Cardiomyocytes	Cell fusion	Reprogram the adult cardiac cells towards a progenitor-like state	Xu X et al., Cell Metab, 2013 [42]

## The role of mitochondrial biogenesis in the multi-directional differentiation of the MSCs

**Fig. 1:**
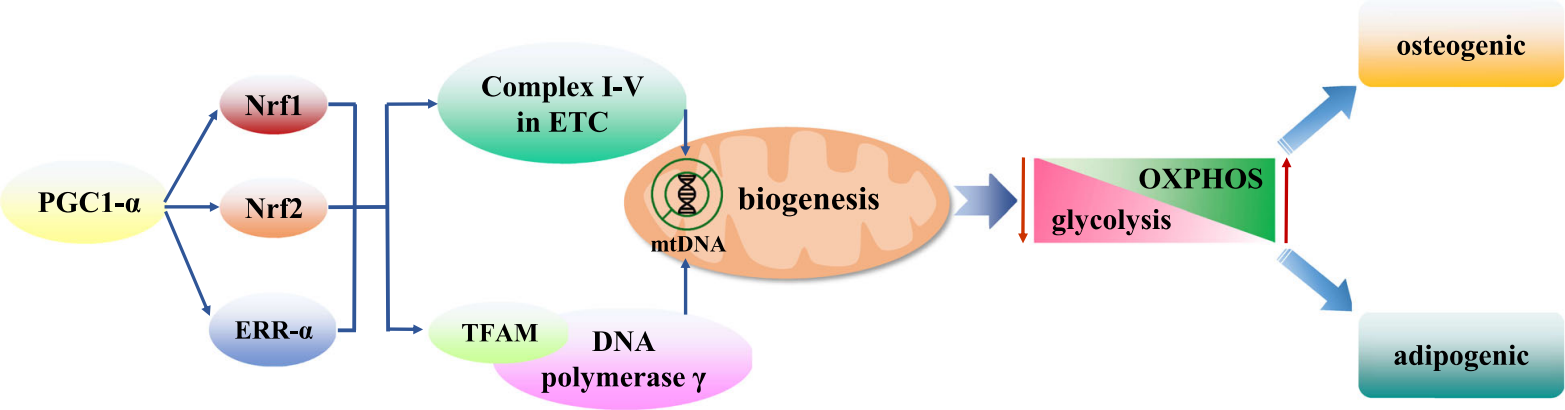
The role of mitochondrial biogenesis in the differentiation of MSCs. The biogenesis of mitochondria is controlled by PGC-1α, followed by the activation of Nrf1, Nrf2, and ERR-α, then activates TFAM, which coordinates with the DNA polymerase γ, thus promoting mitochondrial DNA replication. And Nrf1, Nrf2, and ERR-α also activate the Complex I-V in ETC, thus promoting mitochondrial DNA replication. The activation of mitochondrial biogenesis leads to glycolysis weakened and OXPHOS enhanced, which give rise to the osteogenic and adipogenic differentiation of MSCs. ERR-α, Oestrogen-related receptor-α. ETC, Electron transport chain. MSCs, Mesenchymal stem cells. Nrf1, Nuclear respiration factor 1. Nrf2, Nuclear respiration factor 2. OXPHOS, Oxidative phosphorylation. PGC-1α, PPARγ coactivator-1α. TFAM, Mitochondrial transcription factor A

Mitochondrial biogenesis is controlled by PGC-1α that further activates the expression of nuclear respiration factors (Nrf1 and Nrf2) and oestrogen-related receptor-α (ERR-α), which activate mitochondrial transcription factor A (TFAM) to coordinate with the DNA polymerase γ and promote mtDNA replication [44]. In addition, Nrf1, Nrf2, and ERR-α can also bind to promoter regions of nuclear genes which encode the subunits of five complexes (Complex I-V) in mitochondrial electron transport chain (ETC), thereby regulating mtDNA replication [45]. During the differentiation of the MSCs, the biogenesis of mitochondria increases, leading to an increase in the number of mitochondria in the differentiated cells. For example, after an osteogenic induction of the BMSCs, the levels of proteins involved in mitochondrial biogenesis, such as PGC-1α, TFAM, DNA polymerase γ, and protein subunits of Complex III-V in ETC, increase as well [46]. Moreover, during an adipogenic differentiation of the BMSCs, the expression level of the outer mitochondrial membrane protein, TOM20, increases significantly along with the increase in the number of mitochondria as confirmed via staining [24]. In addition, during the differentiation of the BMSCs into hepatocytes, the expression of several mitochondrial proteins and biogenesis regulators increases as well, such as PGC-1α; OXPHOS activity, capacity, and efficiency; ratio of mitochondria to cytoplasm; and the mtDNA content in the differentiated cells [47]. Similarly, an osteogenic differentiation of the BMSCs and UCMSCs is accompanied by mitochondrial biogenesis that is characterised by an increase in the expression of regulatory factors that induce mitochondrial biogenesis, mtDNA copy number, cristae development, and expression and activity of the OXPHOS complex [19]. In summary, these studies indicate that the differentiation of the MSCs is often accompanied by mitochondrial biogenesis which is regulated by PGC-1α; and caused glycolysis weakened and OXPHOS enhanced, in turn generating enough energy to meet the metabolic needs of the MSCs (Fig. 1).

## The role of mitochondrial dynamics in the regulation of MSC functions

**Fig. 2:**
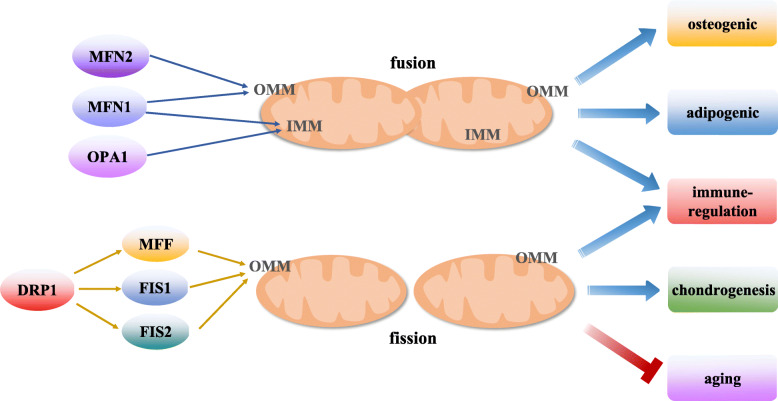
The role of mitochondrial dynamics in the function regulation of MSCs. Mitochondrial fusion is activated during the adipogenic and osteogenic differentiation, and immune regulation of the MSCs. The process of mitochondrial fusion involves the fusion of OMM and IMM. MFN1 and MFN2 mediate the fusion of the OMM, while the OPA1 protein and MFN1 mediate the fusion of the IMM. Moreover, mitochondrial fission is enhanced during the chondrogenic differentiation and immune regulation of the MSCs and protects the MSCs against ageing. And DRP1 regulates the mitochondrial fission, and the receptors, such as MFF, FIS1, and FIS2, are recruited into the OMM and induce mitochondrial contraction and fission. DRP1, Dynamin-related protein 1. FIS1, Fission 1. FIS2, Fission 2. IMM, Inner mitochondrial membrane. MFF, Mitochondrial fission factor. MFN1, Mitofusin 1. MFN2, Mitofusin 2. MSCs, Mesenchymal stem cells. OMM, Outer mitochondrial membrane. OPA1, Optic atrophy 1

Mitochondrial dynamics mainly include the fusion and fission of the mitochondria and mostly depend upon the biological processes, such as apoptosis, calcium homeostasis, and ATP production [14]. Mitochondrial fusion includes the fusion of the inner mitochondrial membrane (IMM) and outer mitochondrial membrane (OMM). The dynamic protein-related GTPases, mitofusin 1 and 2 (MFN1 and MFN2, respectively), mediate the fusion of the OMM, while optic atrophy 1 (OPA1) and MFN1 mediate the fusion of the IMM. Some other proteins also participate in mitochondrial fusion, including prohibitin that regulates OPA1 [48]. In contrast, the mitochondrial fission is mainly regulated by the dynamin-related protein 1 (DRP1) that induces mitochondrial contraction and fission when receptors, such as mitochondrial fission factor (MFF), Fission 1 (FIS1), and Fission 2 (FIS2), are recruited to the OMM. Moreover, multiple post-translational modifications are also involved in the regulation of the mitochondrial dynamics [49, 50]. During the differentiation of the MSCs, the mitochondrial dynamics change; for instance, Forni et al. reported that in the early stage of adipogenic and osteogenic differentiation, the content of citrate synthase in mouse MSCs significantly increases, MFN1 and MFN2 are upregulated, and the mitochondria elongate; these indicate the occurrence of mitochondrial fusion during adipogenesis and osteogenesis. Furthermore, during chondrogenesis, the expression of *DRP1*, *FIS1*, and *FIS2* increases; the knockout of these genes results in the loss of the chondrogenic differentiation ability of the MSCs in mice [30]. Moreover, melatonin can promote the mitochondrial dynamics and metabolism of the BMSCs, enhance the functions of the mitochondria, and protect the BMSCs from excessive ageing in mice with chronic kidney disease [51]. Further, the mitochondrial dynamics of amniotic membrane-derived MSCs can affect the immune regulatory function of the MSCs [52]. In conclusion, the above studies suggest that the mitochondrial dynamics play a critical role in regulating the multi-directional differentiation, ageing, and immune regulation of MSCs via different mechanism (Fig. 2).

## The role of mitophagy in the regulation of the MSC functions

**Fig. 3:**
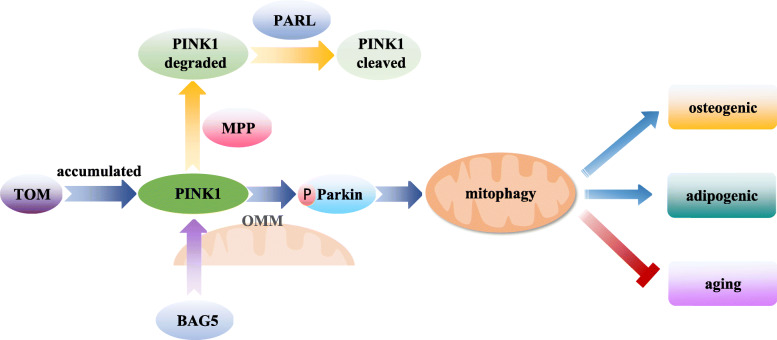
The role of mitophagy in the function regulation of MSCs. During mitochondrial stress or injury, PINK1 accumulates on the OMM through TOM, which leads to Parkin recruitment through PINK1-dependent phosphorylation, subsequently caused the formation of mitochondrial phagosomes, and finally induces the mitophagy. Mitophagy is activated during MSC osteogenic and adipogenic differentiation, which is essential to inhibit ageing in MSCs, while PINK1 is degraded by MPP and subsequently cleaved by PARL. And BAG5 can maintain the function of PINK1. BAG5, BCL2 associated athanogene 5. MPP, Matrix processing peptidases. MSCs, Mesenchymal stem cells. OMM, Outer mitochondrial membrane. PARL, Presenilin-associated rhomboid like. PINK1, PTEN-induced kinase 1. TOM, Translocase of the outer membrane

Mitophagy is a process in which mitochondrial membrane depolarisation stabilises PTEN-induced kinase 1 (PINK1) on the OMM during mitochondrial stress or injury. PINK1 accumulates on the OMM through the translocase of the outer membrane (TOM), which leads to the recruitment of E3 ubiquitin ligase Parkin through the PINK1-dependent phosphorylation and a subsequent formation of mitochondrial phagosomes [53, 54]. The purpose of mitophagy in the MSCs is to eliminate the damaged or dysfunctional mitochondria and control their number [55]. While under normal conditions, PINK1 is continuously targeted to the mitochondria through a mitochondrial targeting sequence, degraded by matrix processing peptidases (MPP) and subsequently cleaved by presenilin-associated rhomboid like (PARL), a protease in the mitochondrial inner membrane. Cleaved PINK translocates to the cytosol and is degraded by the proteasome [56]. Nuschke et al. reported that the accumulation of LC3-II protein, a marker of mitophagy activation, is associated with osteogenic differentiation; this suggests that mitophagy is activated during BMSC differentiation [57]. Consistent with this conclusion, Song et al. also reported that the BMSCs promoted adipogenic differentiation through mitophagy. The addition of mitophagy inhibitors, chloroquine and 3-methyladenine, could inhibit the adipogenic differentiation of the BMSCs, indicating that MSC differentiation is closely related to mitophagy as well [58]. In some diseases and in the ageing process, the accumulation of damaged mitochondria can lead to the deterioration of the stem cell properties. When dysfunctional mitochondria accumulate in the MSCs and do not undergo mitophagy, they may directly affect the activity and function of the stem cells and hinder tissue renewal and regeneration [59]. In addition, the reduction of BCL2-associated athanogene 5 (BAG5), a direct target of miR-155-5p (the most important miRNA in inflammation and ageing tissues), can lead to the dysregulation of PINK1, and thereby destroy the mitophagy of the BMSCs and lead to cell ageing [60]. Therefore, mitophagy is regulated by PINK1-Parkin pathway and activated during MSC osteogenic and adipogenic differentiation, and the restoration of the mitophagy function of the damaged mitochondria is essential to maintain the multi-directional differentiation and self-renewal and inhibit ageing in the MSCs (Fig. 3).

## The role of mitochondrial energy metabolism in the regulation of MSC function

### Role of glycolysis and oxidative phosphorylation in MSCs

Most studies have reported that stem cells mainly rely on glycolysis for metabolism [13]. This may be because higher glycolysis rates coupled with reduced OXPHOS are necessary for the MSCs to provide the co-factors and substrates of the biosynthetic reactions required for their proliferation [14]. In addition, anaerobic metabolism may help in avoiding oxidative damage caused by the ROS produced by mitochondria, and avoid damage to the genetic material and cellular components [61]. Meanwhile, the MSCs have also been considered to rely on anaerobic energy metabolism because they are always isolated from the source tissues in a hypoxic microenvironment, and their dependence on glycolysis may be a long-term evolutionary adaptation of the stem cells to their hypoxic niche. In addition, the MSCs in damaged tissues or transplanted MSCs are usually exposed to hypoxic environments; this interferes with the aerobic metabolism in cells, and consequently, they utilise anaerobic glycolysis to provide most of the energy required for the cellular functions [46]. However, when the BMSCs are cultured in normoxia (21% O_2_), OXPHOS can be effectively utilised under these culture conditions. Interestingly, a 21% O_2_ level increases the ageing of the BMSCs when compared with the physiological oxygen level (5% O_2_). This indicates that a hypoxic environment is conducive to glycolytic metabolism and may protect the MSCs from ageing [62]. Therefore, the MSCs mostly carry out cellular respiration and energy metabolism through glycolysis, perhaps because it is beneficial for their proliferation, helps avoid oxidative damage, or as they are located in a hypoxic niche.

During MSC differentiation, the energy acquisition pathway of the MSCs changes from glycolysis to mitochondrial oxidative metabolism that generates a large amount of energy through mitochondrial OXPHOS to meet the needs of differentiation, while mitochondrial dysfunction impairs this process [25, 30, 63]. The upregulation of mitochondrial biogenesis and aerobic metabolism are characteristics of MSC differentiation [46]. In undifferentiated BMSCs, HIF-1α, which promotes glycolytic gene expression, is highly expressed, while during the osteogenesis induction of the BMSCs, the HIF-1α gene expression, glycolytic enzyme expression, and lactate production decrease and the oxygen consumption rate (OCR) and ATP production significantly increase [24, 25]. Similarly, the oxygen consumption, mitochondrial biogenesis, and respiratory enzyme complex activity are significantly increased during the adipogenic differentiation of the BMSCs [24, 64]. The mitochondrial biogenesis and OCR of mouse skin MSCs (msMSCs) increases during adipogenesis, suggesting that the undifferentiated MSCs transition from glycolysis to OXPHOS during adipogenic differentiation [30]. Furthermore, the glycolysis-dependent MSCs require OXPHOS activity for their differentiation [65]. This indicates that the MSC differentiation is accompanied by an attenuation of glycolysis in mitochondria and the enhancement of the TCA cycle and OXPHOS; moreover, this bioenergy conversion plays a crucial role in the differentiation of MSCs (Table 2).

**Table 2:** Energy metabolic pathways of the MSCs during differentiation

MSC type	Condition	Energy metabolism pathway	Reference
BMSCs	Undifferentiated	Glycolysis	Zhang Y et al., PLoS One, 2013 [24]; Shum LC et al., Stem Cells Dev, 2016 [25]
BMSCs	Osteogenic differentiation	OXPHOS	Shum LC et al., Stem Cells Dev, 2016 [25]
Mouse skin MSCs	Adipogenic differentiation	OXPHOS	Forni MF et al., Stem Cells, 2016 [30]
BMSCs	Adipogenic differentiation	OXPHOS	Zhang Y et al., PLoS One, 2013 [24]; Tahara EB et al., Free Radic Biol Med, 2009 [64]

After the addition of an HIF-1 agonist, dimethyloxalylglycine, the *ALP* gene expression decreases, extracellular acidification rate increases, and OCR decreases; this indicates that the mitochondrial glycolysis was enhanced, and OXPHOS and osteogenic differentiation were inhibited [25]. Similarly, Shares et al. showed that the treatment of C3H10T1/2 MSCs with an OXPHOS inhibitor, antimycin A, could block the connection between mitochondrial activity and β-catenin acetylation, thereby reducing their osteogenic differentiation potential [66]. Moreover, inducing hypoxia or specific knockout of TFAM, to inhibit the mitochondrial electron transport chain and reduce mitochondrial aerobic respiration, could significantly inhibit the adipogenic differentiation of the BMSCs, indicating that the energy metabolism of mitochondria could regulate the differentiation of the MSCs [24]. In addition, treating AD-MSCs with mangiferin, a class of flavonoids extracted from mango, could improve the mitochondrial respiratory function by increasing the expression of mitochondrial OCR and OXPHOS-related proteins, thereby inducing the AD-MSCs to differentiate into brown adipocytes and improve obesity [67]. Rab27b, a member of the small GTPases family, can increase the OXPHOS in cardiac mesenchymal stem cells (C-MSCs) and significantly reduce mitochondrial glycolysis; consequently, this maintains the fatty acid oxidative metabolism of the C-MSCs, suggesting that regulatory genes and drugs may ultimately play a therapeutic role by affecting the mitochondrial energy metabolism [68]. However, recent studies have reported that canagliflozin, a therapeutic medicine for type 2 diabetes, can inhibit the activity of glutamate dehydrogenase 1, which interferes with mitochondrial OXPHOS and ATP production; consequently, it inhibits the proliferation and migration of the BMSCs, which may lead to a decline in the tissue repair ability of the transplanted BMSCs. Thus, some compounds may affect the energy metabolism process of the MSCs and produce secondary actions [47]. In conclusion, current studies have confirmed that regulatory genes and compounds can induce a transformation of the mitochondrial energy metabolism and regulate the function of the MSCs (Table 3). Moreover, key enzymes that regulate chromatin (i.e., DNA and histone) and protein modification processes (i.e., acetylation and methylation) rely on mitochondrial metabolic intermediates as co-factors; this suggests that mitochondrial energy metabolism may be a possible method to regulate the stem cell activity for mitochondria [69, 70]. Furthermore, glycolysis and OXPHOS in MSCs are bi-directional and interact with each other; interestingly, the induction of pluripotent stem cells from somatic cells requires a reverse transformation from OXPHOS to glycolysis [71]. A previous study showed that when inflammation impairs the mitochondrial OXPHOS, it activates intra-cellular glycolysis as a temporary solution to maintain the energy supply [72]. In general, mitochondrial energy metabolism not only changes the process of stem cell differentiation, but also plays a significant role in the regulation of the stem cell functions. To conclude, the undifferentiated MSCs are highly dependent on glycolysis for maintenance and self-renewal, while during the initiation of MSC differentiation, the transformation of the metabolic pathways and an upregulation of the mitochondrial functions are essential for a successful differentiation. Finally, inhibition or promotion of the mitochondrial energy metabolism-related factors can affect the differentiation, proliferation, and migration of the MSCs.

**Table 3:** The effects of the functional regulation and mechanisms of different genes and compounds on the function of the MSCs through changes in the mitochondrial energy metabolism pathway

MSC type	Gene/compound	Energy metabolism pathway	Functional regulation and mechanism	Reference
BMSCs	Dimethyloxalylglycine	Increase glycolysis and inhibit OXPHOS	Inhibit the osteogenic differentiation ability of the BMSCs	Shum LC et al., Stem Cells Dev, 2016 [25]
C3H10T1/2 mesenchymal stem cells	Antimycin A	Inhibit OXPHOS	Inhibit the osteogenic differentiation ability of the C3H10T1/2 mesenchymal stem cells	Shares BH et al., J Biol Chem, 2018 [66]
BMSCs	TFAM	Inhibit OXPHOS	Inhibit the adipogenic differentiation ability of the BMSCs	Zhang Y et al., PLoS One, 2013 [24]
AD-MSCs	Mangiferin	Increase OXPHOS	Induce the differentiation of the AD-MSCs into brown adipocyte phenotype and improve obesity	Rahman MS et al., Metabolism, 2020 [67]
C-MSCs	Rab27b	Increase OXPHOS and inhibit glycolysis	Maintain fatty acid oxidative metabolism of C-MSCs	Jin Y et al., Front Cell Dev Biol, 2020 [68]
BMSCs	Canagliflozin	Inhibit OXPHOS	Inhibit the proliferation and migration of the BMSCs, which may lead to the decrease in tissue repair abilities of BMSCs	Wanet A et al., Int J Biochem Cell Biol, 2014 [47]

### The role of MMP in the regulation of MSCs function

MMP is the driving force behind electron flow and ATP production; when OXPHOS is coupled with it, the free energy difference during the electron transfer causes the transmembrane movement of H^+^, leading to the development of an electrochemical proton gradient across the mitochondrial membrane, and then a proton reflux along the gradient to release energy [31]. A recent study has found that in a long-term culture of human placenta-derived mesenchymal stem cells (PD-MSCs), the MMP of the aged PD-MSCs decreased and the mitochondrial volume increased; this indicated that the ageing PD-MSCs had a mitochondrial dysfunction. The study indicated that carnitine palmitoyltransferase 1A (CPT1A), a key rate-limiting enzyme of fatty acid transfer, was overexpressed in the aged PD-MSCs; consequently, an inhibition of CPT1A expression caused changes in the energy metabolism of the PD-MSCs, increased their MMP, and reversed ageing [73]. Similarly, the apoptosis of the BMSCs induced by decitabine (DAC), which plays an important role in cell cycle arrest and cell death induction, is positively correlated with the mitochondrial dysfunction that is caused by a decrease in the MMP. DAC triggers cell damage in a concentration-dependent manner, the greater the concentration of DAC, the more the cell damage. However, after the addition of a strong antioxidant, *N*-acetyl-l-cysteine (NAC), the MMP was restored by inhibiting the generation of ROS in mitochondria. Thus, as DAC-induced apoptosis can be effectively reversed, it indicates that the MMP may be used as one of the features in determining whether MSC function is impaired [27]. Other compounds can restore the function of the MSCs by increasing the MMP. For instance, Lee et al. have reported that melatonin can induce heat shock 70 kDa protein 1L (HSPA1L) to bind to the cellular prion protein (PrP^C^) by upregulating the expression of HSPA1L in the BMSCs. Then, the HSPA1L-PrP^C^ complex binds to COX4IA (a mitochondrial complex IV protein), leading to an increase in the MMP and antioxidant enzyme activity. Further, this protects the MSCs against replicative senescence during ex vivo expansion in clinical applications via mitochondrial quality control and MMP [74]. Meanwhile, the peroxisome proliferator activated receptor γ (PPARγ) agonist, pioglitazone, alleviates the compression-induced MMP decrease in the nucleus pulposus-mesenchymal stem cells (NP-MSCs), protects cell viability, promotes cell proliferation of the NP-MSCs, and alleviates the toxic effects caused by compression [75]. As the mitochondrial energy metabolism of the MSCs is always accompanied by a change in the MMP (Table 4), their functions may be restored by regulating the changes in the membrane potential to avoid the ageing and apoptosis of the MSCs.

**Table 4:** The effects of the functional regulation and mechanisms of different enzymes and compounds on the MSCs through changes in mitochondrial membrane potential

Enzymes/compounds	MSC type	Change in the membrane potential	Functional regulation and mechanism	References
CPT1A	PD-MSCs	Reduce	Overexpression in senescent PD-MSCs leads to cell senescence	Seok J et al., Stem Cell Res Ther, 2020 [73]
Decitabine	BMSCs	Reduce	Apoptosis of BMSCs	Wang L et al., Eur J Pharmacol, 2019 [27]
Melatonin	BMSCs	Increase	Protects the BMSCs from replicative senescence during proliferation	Lee JH et al., Ageing Cell, 2020 [74]
Pioglitazone	NP-MSCs	Increase	Inhibits oxidative stress and mitochondrial apoptosis, and protects the proliferation of NP-MSCs	Hu Y et al., Oxid Med Cell Longev, 2019 [75]

### Effects of ROS and oxidative stress on mitochondria and regulation of the MSC functions

For a long time, ROS have been considered as a cause of cell dysfunction and tissue death due to the destructive oxidation of the intra-cellular components. Excessive ROS can lead to DNA damage, lipid peroxidation, and protein oxidative modification, and thus damage the cell functions [46]. However, with the rise in studies related to mitochondrial metabolism and dysfunction, people have developed a new understanding of the role of ROS as a signalling molecule. Previous research has demonstrated that photobiomodulation can promote the migration of the human gingival mesenchymal cells by promoting the activation of mitochondrial ROS and increasing the phosphorylation levels of c-Jun N-terminal kinase and IκB kinase [76]. In addition, the physiological upregulation of the ROS is necessary for the MSC proliferation, while its inhibition hinders their self-renewal [77]. Therefore, only an un-regulated level of the ROS is harmful, as their normal physiological level is necessary and beneficial for maintaining the functions of the MSCs [78].

Unlike their differentiated states, the MSCs in their undifferentiated states have low levels of intra-cellular ROS and high levels of antioxidant enzymes [79]. Studies have reported that excessive ROS levels can impair the osteogenic differentiation ability of the MSCs [68, 80]. However, in the adipogenic differentiation of the MSCs, the production and increase in the ROS levels is not only a result of the adipocyte differentiation, but also one of the conditions for the adipogenic differentiation of the MSCs. During the adipogenic differentiation of msMSCs, the mitochondrial biogenesis and ROS expression levels are significantly increased. The excessive ROS levels can lead to the activation of a positive feedback of PPARγ, and thereby accelerate the adipogenic differentiation of the msMSCs [30]. Further, the chondrogenic differentiation of the BMSCs is related to the increase in levels of intra-cellular ROS; however, this may lead to oxidative stress, which is not conducive to cartilage regeneration. Thus, reducing the levels of ROS may be an effective way to increase the collagen accumulation [81]. Above all, the ROS levels in the MSCs are different before and after differentiation, and different differentiation directions lead to varying ROS production. Further, age, long-term culture in vitro, presence of H_2_O_2_, and oxidative stress are major factors that induce MSC senescence. Senescence easily increases the ROS levels, changes mitochondrial morphology, reduces antioxidant capacity, and increases the apoptosis rate; an ROS-mediated oxidative stress-induced replicative senescence can lead to cell membrane lipid peroxidation, mitochondrial dysfunction, energy failure, and metabolic disorders [82]. Another study has reported that a long-term culture of AD-MSCs to induce replicative senescence can lead to a decrease in their proliferation, cell cycle arrest, and differentiation by inhibiting ROS-induced c-Maf, which is sensitive to oxidative stress [83]. Similarly, a long-term culture of human umbilical cord blood mesenchymal stem cells (UCB-MSCs) can ultimately induce ageing by activating the p38 MAPK and p53/p21 signalling pathways and enhancing ROS production [84]. Moreover, H_2_O_2_ is also an important factor leading to oxidative stress-induced premature senescence that has been reported to inhibit the MSC proliferation in a concentration-dependent manner, suggesting that ageing is closely related to ROS production [85]. Furthermore, in BMSCs with an iron overload, an increase in the ROS levels can lead to the activation of the AMPK kinase complex, trigger mitochondrial division, and ultimately lead to apoptosis [86]. The above studies indicate that the ROS levels affect the differentiation direction of the MSCs; however, their excessive levels lead to oxidative stress, which may cause mitochondrial dysfunction, apoptosis, and senescence of the MSCs.

To eliminate the increase in the ROS levels and reduce its side effects, some compounds have been found to regulate the ROS levels, and thereby enhance the activities of the MSCs and promote their application in regenerative medicine (Table 5). For instance, Chen et al. have reported that a co-culture of rat BMSCs with Mg, in vitro, can reduce oxidative stress injury, increase antioxidant enzyme activity, maintain redox homeostasis, and increase MMP; this reduces the risk of UV-induced apoptosis to treat diseases caused by oxidative stress injury [87]. The study also reported that 17β-estradiol, an important regulator of energy homeostasis and glucose metabolism, can protect the UCB-MSCs from high glucose-induced mitochondrial ROS production by increasing the nuclear translocation of Nrf2, and thereby protecting the cells from autophagic cell death [88]. Similarly, the PPARγ agonist, pioglitazone, can suppress the compression-induced oxidative stress in the NP-MSCs; this includes decreasing the compression-induced overproduction of ROS, alleviating compression-induced MPP decrease, and thereby protecting cell viability, cell proliferation, and alleviating the toxic effects caused by compression [75]. Furthermore, a combined treatment with NAC and l-ascorbic acid 2-phosphate promotes the growth of human AD-MSCs and suppresses the oxidative stress-induced cell death by enhancing mitochondrial integrity and function in vitro [89]. The above studies suggest that eliminating excessive ROS levels and maintaining the physiological ROS level are essential targets for mitochondrial-related therapy in MSCs.

**Table 5:** The effects of functional regulation and mechanisms of compounds on MSCs through changes in ROS levels and oxidative stress

Therapeutic method/compound	MSC type	Functional regulation and mechanism	References
Mg	Rat bone marrow mesenchymal stem cells	Reduce the risk of apoptosis induced by ultraviolet radiation	Chen Y et al., J Biomed Mater Res A, 2019 [87]
17β-estradiol	UCB-MSCs	Increase the nuclear translocation of Nrf2 to protect UCB-MSCs from high glucose-induced mitochondrial ROS production, and thereby protect the cells from autophagic cell death	Oh JY et al., Free Radic Biol Med, 2019 [88]
Pioglitazone	NP-MSCs	Decrease the compression-induced overproduction of ROS, protect cell viability and cell proliferation of NP-MSCs, and alleviate the toxic effects caused by compression	Hu Y et al., Oxid Med Cell Longev, 2019 [75]
NAC and l-ascorbic acid 2-phosphate	AD-MSCs	Promote the growth of human AD-MSCs and suppress the oxidative stress-induced cell death by enhancing mitochondrial integrity and function	Li CJ et al., Oxid Med Cell Longev, 2017 [89]

### Key factors affecting the mitochondrial energy metabolism regulation in MSCs

#### HIF-1α

Hypoxia-inducible factor-1 (HIF-1) is a heterodimer comprising a structurally stable β-subunit and an active oxygen-regulated α-subunit (HIF-1α). HIF-1α is continuously synthesised and degraded by prolyl hydroxylase under normoxic conditions. As a response to hypoxia, the HIF-1α activation promotes the transition from aerobic respiration to anaerobic glycolysis and inhibits mitochondrial biogenesis [90]. Mahato et al. have reported that the protein kinase C isoform λ/ι (PKCλ/ι), a major regulator of mitochondrial functions, participates in the co-regulation of mitochondrial biogenesis, and stem cell pluripotency and differentiation by activating HIF-1α. Thus, the depletion of PKCλ/ι causes a metabolic transition, increase in glycolysis, and decrease in OXPHOS activity [91]. Furthermore, most MSCs exist in a hypoxic environment, and such long-term hypoxic conditions upregulate the expression of HIF-1α. When compared with normoxic MSCs, the hypoxic MSCs show higher cell viability, lower ROS levels, and higher resistance to oxidative stress; such hypoxia-induced MSCs alleviate early radiation pneumonia and late pulmonary fibrosis, indicating that HIF-1α plays a key role in regulating the stem cell functions [92]. Moreover, HIF-1α improves the ability of the BMSCs to reduce inflammation and inhibit pro-inflammatory T cell generation by regulating the metabolic switch from OXPHOS to glycolysis; this suggests that HIF-1α is a vital effector of BMSC-mediated immune therapy [93]. Meanwhile, the expression of HIF-1α is also important for the quiescence and function of the BMSCs. HIF-1α maintains the expression of pyruvate dehydrogenase kinase, PDK; this prevents the mitochondrial oxidation of acetyl-CoA by inactivating the pyruvate dehydrogenase complex, thus indicating that PDK is beneficial for glycolysis [94]. Similarly, Hsu et al. found that inhibition of HIF-1α by hypoxia or cobalt chloride in BMSCs can prevent osteogenic differentiation [95]. In addition, the presence of ascorbic acid, an antioxidant enzyme co-factor, can increase the activity of HIF-1α hydroxylase, inhibit HIF-1α transcription, cause mitochondrial activation, and then promote BMSC proliferation [96]. In general, HIF-1α inhibits mitochondrial biogenesis and functions, and thus affects the immune regulation, multi-directional differentiation, and proliferation of the MSCs.

#### PGC-1α

PGC-1α is a major regulator of mitochondrial biogenesis, is highly expressed in MSCs when the energy metabolism demand is high, and promotes OXPHOS in the MSCs [97]. The effect of PGC-1α on the osteogenic and adipogenic differentiation in MSCs is opposite; the overexpression of PGC-1α prevents the osteogenic differentiation potential and increases the adipogenic differentiation potential in the BMSCs [98]. In addition, during the adipogenic differentiation, the *PGC-1α* mRNA level increases, mitochondrial mass and mtDNA copy number increase, OXPHOS complex forms, and OXPHOS activity increases; this suggests that an overexpression of PGC-1α can enhance the oxidative metabolism in mitochondria. Moreover, the inhibition of mitochondrial biogenesis by inhibiting PGC-1α may damage the adipose differentiation potential [24]. In addition, PGC-1α can induce antioxidant enzymes, such as SOD2, which regulate the ROS levels [99]. Thus, the above studies indicate that altering the energy metabolism by regulating the expression of PGC-1α could alter the differentiational fate of the MSCs. Furthermore, the study of PGC-1α will provide a new target for mitochondrial research to improve the mitochondrial activity.

#### SIRT

SIRT is a histone deacetylase that can use the mitochondrial metabolite NAD^+^, as a co-factor to catalyse the modification of lysine during malonylation, succinylation, and glutarylation [70]. SIRT is activated when energy or nutrition is insufficient; this triggers the cellular adaptation and thereby improves the metabolic efficiency [100]. SIRT1 is the most widely studied sirtuin and it controls the activities of many transcription factors, such as p53, NF-κB, forkhead box O, and sterol regulatory element binding protein. It is associated with stem cell activation, reprogramming, and autophagy regulation under stress, controls the mitochondrial biogenesis and function, and affects cell senescence [101, 102]. Further, SIRT1 knockdown can promote the adipogenic differentiation and inhibit osteogenic differentiation in the BMSCs; this suggests that SIRT1 plays a role in the fate determination of the MSCs [103]. Meanwhile, SIRT1 can also deacetylate substrates, such as PGC-1α and liver kinase B1 (LKB1). It has been reported that SIRT1 can induce phosphorylation of LKB1 through deacetylation, thus regulating AMPK [104]. In addition, SIRT3, SIRT5, and SIRT7 are also involved in mitochondrial biogenesis and mitochondrial function activation during adipogenic differentiation [105]. In conclusion, sirtuin can activate mitochondrial biogenesis, improve the metabolic efficiency of mitochondria, and affect the ageing and multi-directional differentiation ability of the MSCs.

#### SOD2

SOD2 is a superoxide dismutase that can catalyse the dismutation of superoxide anion radicals to produce O_2_ and H_2_O_2_. SIRT3 is the main deacetylase that activates SOD2 through deacetylation to reduce the ROS levels and enhance osteogenic differentiation potential of the MSCs [106]. The low-dose histone deacetylases inhibitor, trichostatin A, can protect the MSCs against oxidative stress through the SOD2 regulation mechanism [107]. Similarly, the transforming growth factor, TGF-β1, can significantly downregulate the expression of SOD2 and Id1 in the MSCs, and thus increase the levels of the senescence-related genes in a dose-dependent manner [108]. Furthermore, the BMSC-derived isolated mitochondria could enhance the expression of SOD2 and Bcl-2 and inhibit ROS production in vitro [109]. In summary, SOD2 can enhance the osteogenic differentiation potential of the MSCs and protect the stem cells from ageing by scavenging the intra-cellular superoxide free radicals and inhibiting ROS production.

#### AMPK

AMPK is an energy-sensing kinase and play a crucial role in cell metabolism [110]. Studies reported that expression and phosphorylation of AMPK are increased during osteogenesis in stem cells from human exfoliated deciduous teeth (SHEDs), while after the addition of AMPK inhibitors, compound C, the osteogenic differentiation of AD-MSCs is inhibited and the adipogenic differentiation is promoted, indicating that AMPK is an important molecule regulating the osteogenic and adipogenic differentiation of MSCs [111, 112]. LKB1 is an upstream kinase that regulates AMPK, which can be activated by phosphorylation [113]. Nakada et al. reported that inhibition of LKB1 can reduce the number of mtDNA replication, mitochondrial membrane potential, oxidation capacity, and ATP level in cells and reduce the phosphorylation level of AMPK and the expression of PGC-1α. In addition, Chen et al. also reported that AMPK can directly activate PGC-1α by phosphorylation of PGC-1α [104], suggesting that LKB1, AMPK, and PGC-1α are related to each other, which may be one of the signalling pathways regulating the fate of MSCs. In addition, Mammalian target of rapamycin complex 1 (mTORC1) is a downstream target of AMPK. Pantovic et al. reported that AMPK controls the osteogenic differentiation of dental pulp mesenchymal stem cells (DPSCs) through early mTOR inhibition-mediated autophagy and late activation of the Akt/mTOR signalling axis [114]. Moreover, Tormos et al. reported that BMSCs treated with rapamycin, an inhibitor of mTORC1, can decrease the OCR and the intra-cellular ROS level during adipogenic differentiation, suggesting that mTORC1 has a positive regulation on ROS [115]. In summary, AMPK can promote osteogenic differentiation and inhibit adipogenic differentiation of MSCs, which can be activated by LKB1 and reduce ROS level by inhibiting mTORC1.

#### UCP

UCP is located in the inner membrane of mitochondria, which is part of the mitochondrial respiratory electron transport chain, and can reduce the proton gradient, thus reducing the electrochemical potential of mitochondria, slowing down the oxidative phosphorylation process driven by proton gradient, thus hindering the normal production of ATP and converting proton driven into heat [116, 117]. UCP includes UCP1, UCP2, UCP3, UCP4, and UCP5. UCP1 is considered to be the archetypal uncoupling protein, and which is considered as specific marker of brown adipogenesis and increases respiration, oxygen consumption, dissipates energy, and mitochondrial uncoupled respiration by thermogenesis upon activation [118]. It has reported that decreased UCP-1 expression in beige adipocytes from AD-MSCs is associated with mitochondrial ROS accumulation during obesity [119]. Furthermore, during the differentiation of AD-MSCs into brown-like cells, the expression of UCP-1 increased, differentiated cells show a higher energy metabolism compared to undifferentiated mesenchymal cells, indicating that UCP-1 have a significant role in adipogenic differentiation [120]. In addition, UCP2 can be stimulated by PGC-1α with mitochondrial biogenesis and respiration, suggesting that PGC-1α is an upstream kinase that regulates UCP2 [121]. In summary, UCP can convert the proton gradient which is produced by mitochondrial respiratory chain into thermic energy and closely related to adipogenic differentiation of MSCs.

**Fig. 4:**
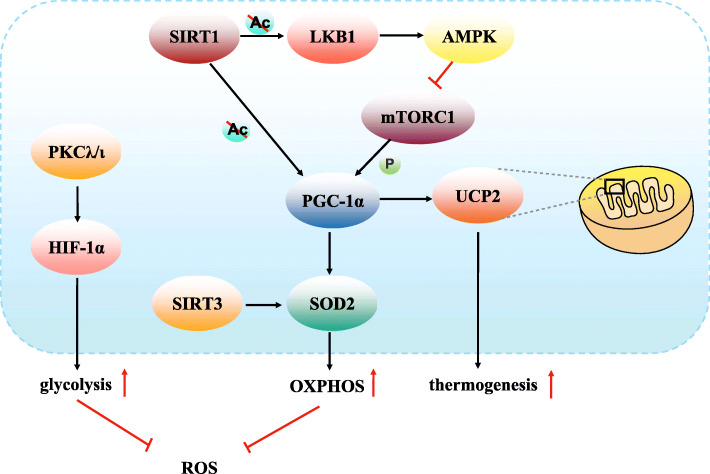
The signalling pathway and key factors which regulated the mitochondrial energy metabolism in MSCs. PKCλ/ι can activate HIF-1α, while HIF-1α promotes glycolysis and decreases ROS level in MSCs. SIRT3 and PGC-1α can induce SOD2, which regulate the ROS levels and promote OXPHOS in MSCs. In addition, PGC-1α can stimulate mitochondrial biogenesis and respiration through induction of UCP2, which can increase thermogenesis upon activation. Mechanismly, SIRT1 induces phosphorylation of LKB1 by deacetylation of LKB1. LKB1 activates AMPK through phosphorylation of AMPK. And mTORC1 is a downstream target of AMPK, which is negatively regulated by AMPK, can phosphorylate PGC-1α. And also SIRT1 can directly deacetylate the phosphorylated PGC-1α. Therefore, LKB1, AMPK, and SIRT1 all participate in the regulation of PGC-1α. AMPK, Adenosine 5′-monophosphate-activated protein kinase. HIF-1α, Hypoxia-inducible factor-1α. LKB1, Liver kinase B1. MSCs, Mesenchymal stem cells. mTORC1, Mammalian target of rapamycin complex 1. OXPHOS, Oxidative phosphorylation. PGC-1α, PPARγ coactivator-1α. PKCλ/ι, Protein kinase C isoform λ/ι. ROS, Reactive oxygen species. SIRT, Sirtuin. SOD2, Superoxide dismutase 2. UCP, uncoupling protein

In conclusion, based on previous studies, we conclude that PKCλ/ι can activate HIF-1α, while HIF-1α promotes glycolysis and decreases ROS levels in MSCs. SIRT3 and PGC-1α can induce SOD2, which regulate the ROS level and promote OXPHOS in the MSCs. In addition, PGC-1α can stimulate mitochondrial biogenesis and respiration through induction of UCP2, which can increase thermogenesis upon activation. Mechanismly, SIRT1 induces phosphorylation of LKB1 by deacetylation of LKB1. LKB1 activates AMPK through phosphorylation of AMPK, and mTORC1 is a downstream target of AMPK, which is negatively regulated by AMPK and can phosphorylate PGC-1α. And also SIRT1 can directly deacetylate the phosphorylated PGC-1α. Therefore, LKB1, AMPK, and SIRT1 all participate in the regulation of PGC-1α (Fig. 4).

## Conclusion

Mitochondria are crucial organelles responsible for the energy metabolism in cells. They can affect several MSC functions such as their differentiation, ageing, immune regulation, apoptosis, proliferation, migration, and chemotaxis. Many studies have reported that mitochondrial morphology, distribution, transfer, biogenesis, dynamics, mitophagy, membrane potential, and ROS production play an important role in maintaining the functions of MSCs in local injury tissues. Further studies have shown that the function of MSCs can be regulated by key effect factors of mitochondrial energy metabolism, such as HIF-1α, PGC-1α, sirtuin, SOD2, AMPK, and UCP. In addition, mitochondrial biogenesis and mitochondrial energy metabolism are closely related to the differentiation process of MSCs. Meanwhile, the united signalling pathway analysis indicates that PGC-1α, which regulates mitochondrial biogenesis, is regulated by SIRT1, mTORC1, and AMPK, but also the upstream component of UCP2, suggesting that PGC-1α is a key factor affecting mitochondrial function. Therefore, how to regulate the level of PGC-1α and then regulate the function of mitochondria can be further explored. Moreover, with the ageing problem becoming more and more serious, how to control the biogenesis, dynamics, mitophagy, and membrane potential of mitochondria to eliminate the damage of mitochondria, prevent MSCs from ageing, and promote tissue remodelling, so as to prevent the ageing of the organism, is also one of the questions that need to be explored. In conclusion, the fate of MSCs can be affected by the change of the energy metabolism and can also be decided by the key factors which regulated the mitochondrial energy metabolism in MSCs. Therefore, how to control the mitochondrial energy metabolism for regulating the proliferation, differentiation, and other functions of MSCs need to be further explored.

Nevertheless, techniques such as mitochondrial transplantation are less operable, mitochondria transfer by autologous MSCs are difficult, the mechanisms and signal targets of mitochondrial energy metabolism in MSCs are unclear, and the effects of modes of mitochondrial energy metabolism on the MSCs are indistinct; all these factors hinder the improvement in MSC functions and their applications in regenerative medicine. Thus, regulating the mitochondrial function in MSCs, improving mitochondrial quality, and exploring mitochondria-related agents may be effective strategies to control and activate the functions of MSCs and employ them in regenerative medicine and treatment of ageing-related diseases. So improving the operability of mitochondrial transplantation, achieving mitochondrial transfer mediated by autologous MSCs, and clarifying the mechanisms, signal targets, and modes of action of mitochondrial energy metabolism in MSCs need to be further performed. These will provide the theoretical basis and candidate targets for promoting MSC function, tissue regeneration, and disease treatment based on mitochondrial function and energy metabolism regulation.

## Data Availability

The data supporting the conclusions of this article are all online.

## Abbreviations

AD-MSCs: Adipose-derived mesenchymal stem cells
ALL: Acute lymphoblastic leukaemia
ARDS: Acute respiratory distress syndrome
AMPK: Adenosine 5′-monophosphate-activated protein kinase
ATP: Adenosine triphosphate
BAG5: BCL2-associated athanogene 5
BMSCs: Bone marrow mesenchymal stem cells
C-MSCs: Cardiac mesenchymal stem cells
CPT1A: Carnitine palmitoyltransferase 1A
DAC: Decitabine
DPSCs: Dental pulp mesenchymal stem cells
DRP1: Dynamin-related protein 1
ER: Endoplasmic reticulum
ERR-α: Oestrogen-related receptor-α
ETC: Electron transport chain
EVs: Extracellular vesicles
FIS1: Fission 1
FIS2: Fission 2
GA: Golgi apparatus
GJs: Gap junctions
HIF-1: Hypoxia-inducible factor-1
HIF-1α: Hypoxia-inducible factor-1α
HSPA1L: Heat shock 70 kDa protein 1L
IMM: Inner mitochondrial membrane
iPSC: Induced pluripotent stem cell
LKB1: Liver kinase B1
MFF: Mitochondrial fission factor
MFN1: Mitofusin 1
MFN2: Mitofusin 2
MitoT: MSC-mediated transfer of mitochondria
MMP: Mitochondrial membrane potential
MPP: Matrix processing peptidases
MSCs: Mesenchymal stem cells
msMSCs: Mouse skin MSCs
mtDNA: Mitochondrial DNA
mTORC1: Mammalian target of rapamycin complex 1
NAC: *N*-acetyl-l-cysteine
NADH: Nicotinamide adenine dinucleotide
NP-MSCs: Nucleus pulposus-mesenchymal stem cells
Nrf1: Nuclear respiration factor 1
Nrf2: Nuclear respiration factor 2
OCR: Oxygen consumption rate
OMM: Outer mitochondrial membrane
OPA1: Optic atrophy 1
OXPHOS: Oxidative phosphorylation
PARL: Presenilin-associated rhomboid like
PDK: Pyruvate dehydrogenase kinase
PD-MSCs: Placenta-derived mesenchymal stem cells
PGC-1α: PPARγ coactivator-1α
PINK1: PTEN-induced kinase 1
PKCλ/ι: Protein kinase C isoform λ/ι
PPARγ: Peroxisome proliferator activated receptor γ
PrP^C^: Cellular prion protein
RA: Rheumatoid arthritis
ROS: Reactive oxygen species
SHEDs: Stem cells from human exfoliated deciduous teeth
SIRT: Sirtuin
sMSCs: Synovial MSCs
SOD: Superoxide dismutase
SOD2: Superoxide dismutase 2
TCA cycle: Tricarboxylic acid cycle
TFAM: Mitochondrial transcription factor A
TNTs: Tunnelling nanotubes
TOM: Translocase of the outer membrane
UCB-MSCs: Umbilical cord blood mesenchymal stem cells
UCMSCs: Umbilical cord-derived mesenchymal stem cells
UCP: Uncoupling protein
